# Characteristics and Outcomes of Cases of Children and Adolescents With Pediatric Inflammatory Multisystem Syndrome in a Tertiary Care Center in Mexico City

**DOI:** 10.3389/fped.2022.849388

**Published:** 2022-05-09

**Authors:** Ricardo Gil Guevara, María de Lourdes Marroquín Yáñez, Rodolfo Norberto Jiménez-Juárez, Víctor Olivar Lopez, Adrián Chávez Lopez, Juan José Luis Sienra Monge, Lourdes Maria del Carmen Jamaica Balderas, Silvia Alexandra Martínez Herrera, Clemen Domínguez-Barrera, Julio Erdmenger Orellana, Horacio Márquez González, Miguel Klünder-Klünder, Jaime Nieto Zermeño, Mónica Villa Guillen, Nadia González García, Maria F. Castilla-Peon

**Affiliations:** ^1^Department of Emergency Medicine, Hospital Infantil de Mexico Federico Gomez Instituto Nacional de Salud, Mexico City, Mexico; ^2^Pediatric Intensive Care Unit, Hospital Infantil de Mexico Instituto Nacional de Salud, Mexico City, Mexico; ^3^Department of Infectious Diseases, Hospital Infantil de Mexico Federico Gomez Instituto Nacional de Salud, Mexico City, Mexico; ^4^Department of Pediatrics, Infectious Diseases Hospital, UMAR National Medical Center “La Raza,” Instituto Mexicano del Seguro Social, Mexico City, Mexico; ^5^Ambulatory Pediatrics, Hospital Infantil de Mexico Federico Gomez Instituto Nacional de Salud Instituto Nacional de Salud, Mexico City, Mexico; ^6^Department of Respiratory Medicine, Hospital Infantil de Mexico Federico Gomez Instituto Nacional de Salud, Mexico City, Mexico; ^7^Department of Cardiology, Hospital Infantil de Mexico Federico Gomez Instituto Nacional de Salud, Mexico City, Mexico; ^8^Department of Clinical Research, Hospital Infantil de Mexico Federico Gomez Instituto Nacional de Salud, Mexico City, Mexico; ^9^Research Division, Hospital Infantil de Mexico Federico Gomez Instituto Nacional de Salud, Mexico City, Mexico; ^10^Hospital Infantil de Mexico Federico Gomez Instituto Nacional de Salud, Mexico City, Mexico; ^11^Neruosciences Research Laboratory, Hospital Infantil de Mexico Federico Gomez Instituto Nacional de Salud, Mexico City, Mexico

**Keywords:** pediatric multisystem inflammatory disease, COVID-19, SARS-CoV-2, children, coronary aneurysms, Mexico

## Abstract

**Background:**

Pediatric inflammatory multisystem syndrome (PIMS) is a complication of severe acute respiratory syndrome coronavirus 2 (SARS-CoV-2) infection in children that resembles Kawasaki syndrome and places them at high risk of cardiorespiratory instability and/or cardiac damage. This study aims to describe the clinical presentation and outcomes of patients with PIMS in Mexico City.

**Methods:**

This was an observational study of children hospitalized for PIMS based on the Centers for Disease Control and Prevention case definition criteria, in a single tertiary care pediatric center in Mexico City between May 1, 2020, and September 30, 2021. Demographic characteristics, epidemiological data, medical history, laboratory tests, cardiologic evaluations, treatment, and clinical outcomes were analyzed.

**Results:**

Seventy-five cases fulfilled the case definition criteria for PIMS [median age: 10.9 years, Interquartile range (IQR): 5.6–15.6]. Fifteen (20%) patients had a severe underlying disease, 48 (64%) were admitted to the intensive care unit, 33 (44%) required invasive mechanical ventilation and 39 (52%) received vasopressor support. The patients were clustered through latent class analysis based on identified symptoms: Cluster 1 had rash or gastrointestinal symptoms (*n* = 60) and cluster 2 were those with predominantly respiratory manifestations (*n* = 15). Two patients (2.7%) died, and both had severe underlying conditions. Five patients (6.7%), all from cluster 1, developed coronary aneurysms.

**Conclusion:**

There were a high proportion of patients with severe respiratory involvement and positive RT-PCR SARS-CoV-2 and very few cases of coronary aneurysms in our study which suggests that a high proportion of the children had severe acute COVID-19. The clinical manifestations and outcomes are comparable to previously reported international studies.

## Introduction

Severe acute respiratory syndrome coronavirus 2 (SARS-CoV-2) infection usually has a mild clinical presentation in children. However, in rare cases, children can be severely affected and have clinical manifestations, different from adults. In April 2020, some reports described a clinical syndrome similar to Kawasaki disease or toxic shock syndrome temporally associated with current or recent SARS-CoV-2 infection in the pediatric population (1). Since then, this syndrome has been recognized worldwide and named pediatric inflammatory multisystem syndrome (PIMS) or multisystem inflammatory syndrome in children (MIS-C) (2–6).

PIMS case definition varies slightly between different health agencies (7–9) and it is likely to change over time. The criteria include fever, inflammatory marker elevation, multisystem organ involvement, evidence of current or recent SARS-CoV-2 infection, and exclusion of alternative diagnoses. Besides the need for hospitalization for life support, an outcome of concern is myocardial and coronary artery involvement similar to those observed in Kawasaki disease (10–12).

PIMS incidence has been estimated to be approximately 3–5 per 10,000 individuals younger than 21 years of age infected with SARS-CoV-2, and Black and Hispanic ethnicities have been associated with a higher incidence (13). Epidemiological description of PIMS has been difficult because of varying awareness of the condition in different clinical scenarios and because of the diversity of clinical manifestations. Besides, there is an overlap between the clinical manifestations of severe acute COVID-19 and PIMS (10). Hence, the current PIMS case definition is purposely broad and unspecific to obtain maximum and comprehensive information about this phenomenon. However, increasing evidence suggests the existence of different phenotypes of PIMS, the more clearly defined of them being a Kawasaki-like syndrome, a toxic shock-like syndrome, and a predominantly respiratory syndrome which might have different pathogenesis and clinical outcomes (14–16).

This study aims to describe the clinical characteristics and outcomes of PIMS cases admitted to a tertiary care pediatric center in Mexico City. A secondary aim was to define potential clinical subgroups of children with PIMS.

## Materials and Methods

### Study Design

This was an observational cohort study of PIMS cases diagnosed in “Hospital Infantil de Mexico Federico Gómez” (Federico Gómez Mexico Children’s Hospital), a tertiary care pediatric facility officially designated to treat patients with severe SARS-CoV-2 infection who are less than 18 years old, and without public or private health insurance in Mexico City.

Probable PIMS cases were identified in the emergency room, transferred from other health care facilities directly to the intensive care unit, or identified by treating physicians in the general hospitalization ward for COVID-19 patients. Cases’ files were evaluated to determine if they met the Centers for Disease Control and Prevention (CDC) PIMS case definition (7) (Table 1). Patients were excluded if they had another plausible explanation for the illness. When there was uncertainty about an alternative diagnosis, the complete case file was reviewed by an expert panel which included an infectious disease specialist and a critical care specialist, until a consensus was reached.

**TABLE 1:** Case definition criteria for PIMS.

Chart 1. Case definition criteria for PIMS	
1. Fever	
2. Elevation of inflammation markers	At least one of the following:
	• C-Reactive Protein (CRP) > 5mg/L
	• Procalcitonin > 0.15 ng/ml
	• Ferritin > 300 mcg/L
	• Fibrinogen > 400 mg/dl
	• D-Dimer > 560 ng/ml
	• Neutrophils > 7500 cells/mcl
	• Lymphopenia: < 2 years: < 4000, 2-3 years: < 3000, > 4 years: < 1500 cells/mcl
	• Albumin < 3 g/dl
3. At least two systems involved	3.1 Cardiovascular. At least one of the following:
	• Required vasoactive drug
	• Left ventricular ejection fraction < 55%
	3.2 Respiratory. At least one of the following:
	• Supplementary oxygen with non-rebreathable mask or higher oxygen concentration device.
	• Oxygen saturation < 90%
	• Pulmonary infiltrates in chest X-Ray or Computed Tomography
	3.3 Neurologic. At least one of the following:
	• Glasgow score < 14 if not sedated
	• Meningismus
	• Seizures
	3.4 Hematologic. At least one of the following:
	• Plateletes < 150 000 cells/mcl
	• Prothrombin time > 14 s
	3.5 Gastrointestinal. At least one of the following:
	• Abdominal pain
	• Vomit
	• Diarrhea
	• Alanine aminotransferase (ALT) > 90 U/L
	3.6 Mucocuteneous. At least one of the following:
	• Rash
	• Extremity edema
	• Desquamation
	• Mucosal inflammation
	• Conjunctivitis
	3.7. Renal
	• Creatinine > twice upper limit of the reference range for age
4. Hospitalization	
5. No alternative diagnosis	• No positive blood culture during the first 48 hours in the hospital
	• In uncertain cases, complete file review and consensus by an expert panel.
6. Present or recent infection by SARS-COV2	At least one of the following
	• Positive RT-PCR^1^ for SARS-CoV-2
	• Positive serology for SARS-CoV-2
	• Contact with a confirmed positive COVID-19 case in the last 4 weeks.

*^1^Reverse transcriptase polymerase chain reaction.*

### Data Collection

Clinical and laboratory data were extracted from the patients’ clinical file and included demographics, underlying medical conditions, clinical manifestations, laboratory values, management, and outcomes. For laboratory values with more than one measurement, the worst value measured within the first 3 days of hospitalization was obtained. Vital support management (respiratory support and vasopressor utilization), intravenous immune globulin, systemic steroids, and anticoagulant therapy were documented as the main therapeutic interventions. Outcomes of interest were intensive care unit (ICU) admission, length of ICU stay and hospitalization, need for invasive mechanical ventilation, vasopressor support, myocardial depression (left ventricular ejection fraction < 55%), coronary aneurysms (coronary artery diameter z-score ≥ 2.5), or dilatation (z-score > 2– < 2.5) on the echocardiographic evaluation performed during hospitalization, and in-hospital mortality. Markers with more than 20% missing data were not analyzed.

For this study, severe respiratory involvement was defined as infiltrates on chest X-ray or computed tomography plus the need for oxygen administration with a non-rebreathable mask or a higher oxygen concentration device. The variable “gastrointestinal symptoms” was defined as the presence of at least one of the following: abdominal pain, diarrhea, or emesis.

The study center has an important regular population of patients with severe underlying diseases. Hence, to have a picture more alike general population, we performed a sub-analysis with the exclusion of cases with severe underlying diseases (i.e., cancer and other forms of immunosuppression diseases, neuromuscular disability, chronic respiratory disease except for asthma, congenital cardiopathies, and chronic kidney failure).

### Statistical Analysis

Descriptive analysis was conducted using STATA v.14.0 (StataCorp, Tx). Categorical variables were reported as frequencies and continuous variables as medians and interquartile ranges (IQR). Analysis was performed on the whole sample and three subgroups. In the first subgroup, cases with severe underlying conditions were excluded. The other two subgroups were identified using latent class analysis (LCA). Two class LCA were conducted using the R software package “poLCA” (17) with 100 iterations to identify the clusters. The fit of each model was assessed using a Bayesian Information Criterion score (BIC). The indicator variables used in the final LCA model were the presence or absence of severe respiratory involvement, rash and gastrointestinal symptoms.

The study was approved by the ethics review board of Federico Gómez Mexico Children’s Hospital (Reg: HIM2020-031), including the publication of de-identified data.

## Results

**FIGURE 1:**
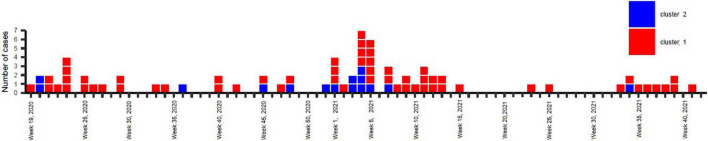
Temporal distribution of PIMS cases by cluster from May 1st, 2020 (Week 19, 2020) to September 30, 2021 (Week 40, 2021).

Reports of one hundred and sixty-seven (167) probable cases of PIMS admitted to the hospital between May 1, 2020, and September 30, 2021, were sent by clinical departments. For reference, during this period, 8,193 real-time polymerase chain reaction (RT-PCR) tests for SARS-CoV-2 were performed in the study center, which yielded 837 positive results. The temporal distribution of cases follows the pattern of the three epidemic peaks in Mexico City during the study period (Figure 1).

From the 167 received reports, 42 were from the emergency department, 34 from the ICU, and 91 from the infectious disease department. Duplicated reports were eliminated (*n* = 23) and 23 reports failed to meet inclusion criteria. Forty-six cases were excluded because either elevated inflammation markers or system failures had an obvious cause other than the exposure to SARS-CoV-2. Of these, in 13 cases an infectious process other than SARS-CoV-2 was identified. In 6 cases, lymphopenia and/or thrombocytopenia were attributed to drugs, mainly antineoplastic chemotherapy. Five cases presented with a new onset malignancy, two had a reactivation of systemic lupus erythematosus, one had a macrophage activation syndrome and one case was diagnosed with Kawasaki syndrome. Eighteen cases were excluded because their comorbid condition explained one of the two organic failures. Of the 46 excluded cases, only the one diagnosed with Kawasaki syndrome received intravenous immune globulin (IVIG).

One included case presented as acute abdomen. Appendicectomy was performed in the referring center and had an unexpected poor evolution after surgery. This case was included because of a lack of data on surgical findings suggestive of abdominal sepsis.

The results for the 75 included cases are as follow: the median [interquartile range (IQR)] age was 10.9 (5.6–15.6) years and 52% were females. A total of 45.3% of the participants had at least one underlying condition, including obesity (25%), cancer (9%), neuromuscular (8%), and respiratory (7%) diseases (Table 2).

**TABLE 2:** Clinical presentation of cases with PIMS.

	Total population (*N* = 75)^1^	Without serious underlying disease^2^ (*N* = 60)^1^	Cluster 1 With GIS^3^ or Rash (*N* = 60)^1^	Cluster 2 Predominant respiratory (*N* = 15)^1^	*p* *
Female sex -no. (%)	39 (52%)	33(55%)	33 (55%)	6 (40%)	0.23
Age–median (IQR)	10.9 (5.6–15.6)	10.6 (5.5–15.0)	10.3 (5.7–13.5)	15.3 (5.4–16.6)	0.08
**Age group–*n* (%)**					
<1 years	5 (6.7%)	5 (8.3%)	4(6.7%)	1 (6.7%)	0.12
1–4 years	11 (17.3%)	9 (15%)	9 (15%)	2 (1.3%)	
5–9 years	13 (17.1%)	11 (18.3%)	12 (0.2%)	1 (6.7%)	
10–14 years	26 (34.7%)	21 (35%)	23 (38.3%)	3 (20%)	
15–18 years	20 (26.7%)	14 (23.3%)	12 (20%)	8 (53%)	
Days of symptoms before admission	7(4–9)	7 (5–9)	6 (4–8)	7 (1–9)	
**Symptom’s onset posterior to June 1, 2021**	11 (14.7%)	10 (16.7%)	10 (16.7%)	1 (6.7%)	0.30
SARS-COV2 testing, No. (%)					
RT- PCR positive	57/71 (80%)	43/56 (76.8%)	44/58 (75.9%)	13/13 (100%)	**0.04**
Serology positive	25/41 (61%)	25/46 (54%)	25/36(69%)	0/5*	**0.01**
N gene Ct (*n* = 38)	31.8 (28.1–36.1)	33.4 (29.4–36.7)	33.9 (30.2–36.7)	25.6 (20.9–30.6)	**0.01**
N gen Ct > 30	24/38 (63.2%)	20/28 (71.4%)	22/28 (78.6%)	2/10 (20%)	**0.002**
**Underlying medical conditions–no. (%)**					
Obesity (BMI > 95°percentile)	18 (24%)	14 (23.0%)	12 (20%)	6 (40%)	0.10
Respiratory	5 (6.7%)	3 (5%)	2 (3.3%)	3(20%)	0.05
Neuromuscular	6 (8.0%)	0	2 (3.3%)	4 (26.7%)	**0.01**
Cardiovascular	3 (4%)	0	2 (3.3%)	1 (6.7%)	0.49
Cancer	7 (9.3%)	0	2 (3.3%)	5 (33%)	**0.003**
Other, immunosuppression	2 (3%)	0	1 (1.7%)	1 (6.7%)	0.32
Other comorbidity	10 (13.3%)	6 (10%)^4^	8 (13.3%)	2 (13.3%)	0.64
At least 1 underlying condition	34 (45.3%)	19 (31.2%)	22 (36.7%)	12 (80%)	**0.003**
**Clinical manifestations–no. (%)**					
Cough	39(52%)	32 (53.3%)	33 (55%)	6 (40%)	0.23
Rash	36 (48%)	33 (55%)	36 (60%)	0	**< 0.001**
Conjunctivitis	39 (50.6%)	39 (52%)	36 (60%)	2 (13.3%)	**0.001**
Diarrhea	33 (44%)	27 (45%)	33 (55%)	0	**< 0.001**
**Emesis**	36 (48%)	33 (55%)	36(60%)	0	**< 0.001**
Abdominal pain	38 (51%)	36 (60%)	38 (63.3%)	0	**< 0.001**
Headache	34(45.3%)	25 (41.7%)	27 (45%)	7 (46.7%)	0.57
Rhinorrhea	24 (32%)	20 (33.3%)	19 (31.7%)	5 (33.2%)	0.56
Respiratory Distress	31 (41%)	24 (40%)	21 (35%)	10 (66.7%)	**0.03**
Lower respiratory symptoms	41 (54%)	31 (51.7%)	29 (48.3%)	12 (80%)	**0.03**
Anosmia/Dysgeusia	7 (9.3%)	6 (10%)	4 (6.6%)	3 (20%)	0.14
Arthralgias/Myalgias	14(18.7%)	11 (18.3%)	13 (21.7%)	1 (6.7%)	0.17
Oxygen Saturation < 90%	30 (40%)	20 (33.3%)	22 (36.7%)	8 (53.3%)	0.19
**Laboratory values within 72 h of admission–median (IQR)/*n* (%)**					
Neutrophil count ×10^6^ cells/mcl	6.1 (1.7–9.2)	7.1 (3.1–10.1)	6–5 (1.9–9.8)	3.1 (0.5–7.1)	**0.04**
Neutrophilia (> 7,500 cells/mm^3^)	29/74 (39.2%)	27/59 (45.8%)	27/59 (45.8%)	2 (13.3%)	**0.02**
Neutropenia (< 1,000 cakes/mm^3^)	10 (13%)	6 (10%)	5/59 (8.5%)	5 (33%)	**0.02**
Lymphocyte count ×10^6^ cells/mm^3^	1.5 (0.5–2.4)	1.6 (0.8–2.1)	1.6 (0.6–2.8)	0.8 (0.4–1.8)	**0.16**
Lymphopenia^5^	43/73 (58.9%)	33/58 (56.9%)	33/58 (56.9%)	10 (66.7%)	0.35
Platelets × 10^3^cells/mm^3^	162 (94–250)	166 (110–271)	162 (105–235)	162 (43–330)	0.82
Fibrinogen, mg/dl	510 (404–668)	558 (431–724)	516 (412–671)	510 (361–669)	0.66
Fibrinogen > 400 mg/dl	52 (69.3%)	44/56 (78.6%)	43 (71.7%)	10 (76.9%)	0.5
Ferritin, mcg/L	617 (407–1370)	559 (380–1,110)	604 (417–1,150)	1,370 (325–6,071)	0.25
Ferritin > 300 mg/dl	53 (70.1%)	41 (68.3%)	46/55 (83.6%)	7/9 (77.7%)	0.49
D-Dimer ng/L	2.6 (1.1–6.5)	2.9(1.2–8.8)	3.5(1.3–9.5)	0.8 (0.5–1.2)	**< 0.001**
D-Dimer > 560 ng/ml (0.56 ng/L)	66/74 (89.2%)	55 (91.7%)	55/59 (93%)	11/15 (73.3%)	**0.05**
C-reactive protein (mg/L)	9.8 (4.5–18.9)	13.5 (6.1–19.9)	13.2 (5.9–19.1)	7.3 (0.7–17.7)	0.36
C-reactive protein > 5 mg/L	47/63 (74.6%)	41/50 (82%)	42/54 (77.8%)	5/9 (55.5%)	0.16
Procalcitonin ng/ml	1.85 (0.5–6.3)	1.8 (0.47–3.84)	2.1 (1.16–6.4)	0.37 (0.12–6.17)	0.12
Procalcitonin > 0.15 ng/ml	55/62 (88.7%)	43/50 (86%)	47/51 (92%)	8/11 (72.7%)	0.1
Albumin < 3 g/dl	45/71 (63.3%)	36/56 (64.3%)	40/57 (70.2%)	5/14 (35.7%)	**0.02**

*^1^N in the column heading was used to calculate proportions if not otherwise specified.*

*For variables with missing data, denominators are reported in each cell.*

*^2^Excluded: cancer, immunodeficiency, neuromuscular, chronic respiratory disease except asthma, congenital cardiopathies, chronic kidney failure.*

*^3^GIS, Gastrointestinal symptoms, includes abdominal pain, diarrhea, emesis.*

*^4^Trisomy 21 (n = 1), Post recent appendicectomy (n = 2), hypothyroidism (n = 2), undernutrition (n = 1).*

*^5^Lymphopenia: (< 2 years: < 4,000, 2–3 years: < 3,000, > 4 years: < 1,500 cells/mcl).*

*O one-sided Fisher exact test/Mann–Whitney test for comparison between cluster 1 and cluster 2. p-values < 0.05 are highlighted in bold.

In addition to fever, the most common clinical manifestations were cough (52%), conjunctivitis (50.6%), abdominal pain (51%), rash (48%), headache (45.3%), and diarrhea (44%). The median (IQR) duration of symptoms before admission was 7 (4–9) days. About 51% had lower respiratory symptoms, 85% had pulmonary infiltrates on radiograph or computed tomography, and 65.3% required oxygen administration with a non-rebreathable mask, high flow nasal cannula, or mechanical ventilation. Vasopressor support was used in 52% of the cases, and about 40% had a left ventricular ejection fraction < 55% on echocardiography. Median values for markers of inflammation, coagulation, and system damage are summarized in Table 2.

Three subgroups were analyzed independently. In the first subgroup (*n* = 60), 15 cases with severe underlying disease were excluded. Clinical manifestations and outcomes did not differ significantly from those observed in the total sample (Tables 2, 3).

**TABLE 3:** Treatment and outcomes of cases with PIMS.

	Total sample (*N* = 75)^1^	Without serious underlying disease (*N* = 60)^1^	Cluster 1 With GIS or rash (*N* = 60)^1^	Cluster 2 Predominant respiratory (*N* = 15)^1^	p*
**Treatment–no. (%)**					
Intravenous immune globulin	34(45.3%)	32(53.3%)	33 (55%)	1 (6.7%)	**0.01**
Systemic steroids	48 (64.0%)	43 (71.7%)	44 (73.3%)	4 (26.7%)	**0.001**
Anticoagulation therapy	47 (62.7%)	40 (66.7%)	41 (68%)	6 (40%)	**0.04**
Maximum ventilatory support needed–no (%)					
Non-rebreather mask	8 (10.7%)	7 (11.7%)	1 (1.7%)	1 (6.7%)	
High flow nasal cannula	4 (5.3%)	2 (3.3%)	2 (3.3%)	2 (13.3%)	
Non-invasive mechanical ventilation	4 (5.3%)	4 (6.7%)	4 (6.7%)	0	
Invasive mechanical ventilation	33 (44%)	25 (41.7%)	25 (41.7%)	9 (60%)	0.16
Any infiltrates in chest Rx or CT	60/71 (85%)	48/56 (85.7%)	45/56 (80%)	15 (100%)	0.06
Severe respiratory involvement^2^	28 (37.3%)	21 (35%)	19 (31.7%)	9 (60%)	**0.04**
**Cardiovascular involvement–no (%)**					
LVEF^3^ < 55%	30 (40.0%)	24 (40%)	22 (36.7%)	8 (53.3%)	0.18
LVEF < 45%	6 (8.0%)	5 (8.3%)	6 (10%)	0	0.25
LVEF < 35%	0	0	0	0	
Coronary aneurysm (z score > 2.5)	5 (6.7%)	5(8.3%)	5 (8.3%)	0	0.32
Coronary dilatation (z score > 2–< 2.5)	0	0	0	0	
Vasopressor support–no (%)	39 (52.0%)	30 (50%)	32 (53.3%)	7 (46.7%)	0.43
Hematologic involvement	50 (66.7%)	39 (65%)	39 (65%)	12 (80%)	
Thromboplastin time > 14 s	37/72 (51.5%)	30 (50%)			0.26
Platelets count < 150 000 cell/mcl	29 (38.7%)	21 (35%)	24 (40%)	6 (40%)	1.0
Neurologic involvement ^4^	5 (6.7%)	5 (8.3%)	5 (8.3%)	0	0.32
Gastrointestinal involvement	60 (80%)	51 (85%)	57 (95%)	3 (20%)	**< 0.001**
Alanine Aminotransferase (ALT) > 90 U/L	21/72 (29.2%)	16/58 (27.6%)	17/58 (29.3%)	4/14 (28.5%)	0.62
Mucocutaneous involvement	64 (85.3%)	39 (65%)	58 (96.7%)	6 (40%)	**< 0.001**
Acute Kidney Injury, stage 2 ^5^	19/74 (25.7%)	26.7%	19 (31.7%)	0	**0.01**
Clinical outcomes- no. (%)/median (IQR)					
ICU^6^ admission	48 (64%)	37 (61%)	38 (63%)	10 (66.7%)	0.53
Length of ICU stay-d (*n* = 48)	5 (3–8)	4(3–8)	4.5 (3–8.3)	7.5 (3.5–13.5)	0.23
Length of Hospital stay days	7 (4–13)	7(4–11)	7 (4–12)	10 (5–17)	0.31
Died	2 (2.7%)	0	1 (1.7%)	1 (6.7%)	0.36

*^1^N in the column heading was used to calculate proportions if not otherwise specified.*

*For variables with missing data, denominators are reported in each cell.*

*^2^Rx: chest X-ray, CT: Computed tomography.*

*Severe respiratory involvement was defined as oxygen requirement and infiltrates in thoracic imaging.*

*^3^LVEF = Left ventricular ejection fraction.*

*^4^Neurologic involvement: seizures (n = 3), optic neuritis (n = 1), meningismus (n = 1), headache not included.*

*^5^Acute Kidney Injury (AKI) was defined as an increase greater than two times the upper limit of the reference range for gender and age.*

*^6^ICU, intensive care unit.*

*Comparison Cluster 1 vs. cluster 2 with Fisher exact test/Mann–Whitney test. p-values < 0.05 are highlighted in bold.

The second (*n* = 60) and third (*n* = 15) subgroups were derived from LCA which identified two classes of patients: cluster 1 (*n* = 60) comprised of patients with rash and/or gastrointestinal symptoms, and cluster 2 (*n* = 15) comprised of those with predominantly cardiorespiratory involvement, without a rash or gastrointestinal symptoms. Despite the small number of cases in cluster 2, some differences were evident: cluster 2 cases were more prone to having a positive RT-PCR test (100 vs. 76%, *p* = 0.04), lower median cycle threshold (Ct) values (33.9 vs. 25.6, *p* = 0.01), and less likely to have a positive serology test (0 vs. 54%, *p* < 0.001). An underlying medical condition was also more frequent in cluster 2 (80 vs. 36.7%, *p* = 0.003), especially a severe one (47 vs. 13%, *p* = 0.008) such as cancer, neuromuscular disability, or a chronic respiratory condition (Table 2). Cluster 1 patients tended toward higher levels of inflammation markers, though the difference was statistically significant for only the neutrophil count and D-Dimer concentration.

With regards to treatment, 45.3, 64, and 63% of the cases received intravenous immune globulin, systemic steroids, and anticoagulation therapy, respectively, which was more frequent in the cluster 1 group (Table 3). For outcomes, two (2.7%) patients died and they both had severe underlying diseases; the first patient had a medulloblastoma and died 2 weeks after the antineoplastic chemotherapy dose while the second one had chronic kidney failure and was on renal replacement therapy. One death occurred in each cluster group. About 44% of the cases needed mechanical ventilation, 52% required vasopressor support while 40% had a depressed ventricular function (Table 3).

All cases had an echocardiographic evaluation during hospitalization. Five cases (6.7%) had coronary aneurysms and notably, all these cases were in cluster 1. Myocardial involvement (left ventricular ejection fraction < 55%) was observed in 30 (40%) of the cases. ICU admission occurred in 64% of the cases with a median (IQR) length of ICU stay of 5 (3–8) days and a length of hospital stay of 7 (4–13). There was a tendency of cluster 2 patients to remain longer in ICU and the hospital (Table 2).

The third epidemic wave started at the beginning of June 2021 (18) and was attributed to the Delta variant of SARS-CoV-2 (19). Eleven of the analyzed PIMS cases occurred in this “Delta wave.” An exploratory analysis showed that these cases had a lower frequency of UTI admission (36.4 vs. 68.8%, *p* = 0.05) than those presenting before June 2021, with no deaths.

## Discussion

To our knowledge, the present study described the largest cohort of patients with PIMS in the Mexican population to date. Most clinical characteristics and outcomes were similar to those described in large international reports. The median age of our sample was higher than most of these previous reports. The prevalence of underlying medical conditions, including obesity, was similar to the reported data in the United States and Latin America (10, 16, 20–22), except for cancer which was relatively more frequent in our total sample, reflecting the characteristics of the population regularly attending the study site. Cardiac dysfunction, vasopressor requirements, rash, gastrointestinal symptoms, ICU admissions, and lengths of hospital and ICU stay were comparable to those reported elsewhere. The death rate of 2.7% (binomial 95% confidence interval: 0.3–9.3%) was also consistent with previous reports (3, 5, 10, 14, 20, 23).

Our population had a relatively high frequency of lower and serious respiratory involvement in comparison with most of the other studies. Besides, in our sample, serology was performed in only 55% of the cases, so the evidence of SARS-CoV-2 infection in our population was mostly by RT-PCR (80% positivity rate). These findings point toward a higher proportion of acute severe SARS-CoV-2 infection vs. a theoretical late-onset post-infectious syndrome compared to other case series where respiratory involvement and RT-PCR positive rate was lower (3, 5, 10, 14, 20). Current PIMS diagnostic criteria do not exclude acute severe COVID-19; this overlap between both clinical presentations has been previously discussed (10, 16).

On the other hand, the frequency of coronary aneurysms in our study was relatively low (6.7%). The frequency of aneurysms varied, ranging from 6.7 to 23.3% in previous studies (14, 20, 21, 23). This probably reflects the lack of specificity in the PIMS case definition criteria and the variability of the cut-point for some inflammatory markers used in different reports. Most case series are derived from active or passive surveillance, either retrospectively or prospectively. Many of them do not inform a cut-point for inflammatory markers concentration, or clinical symptoms and the upper limit of the normal reference range may have been used. This probably explains the high frequency of coronary aneurysms (23.3%) found by Flood et al. in the United Kingdom. Their study had a very high cut-point of 100 mg/L for C-reactive protein and required “acute abdomen” and very specific dermatological findings, in addition to “abdominal pain” or “rash” as criteria for gastrointestinal or mucocutaneous involvement, respectively (14).

While clinical and epidemiological studies point toward the existence of at least two different clinical presentations of severe illness associated with SARS-CoV-2 infection (10, 14–16, 24), a clear-cut distinction between the clinical and physio- pathological features of each one has remained elusive. On the one hand, severe COVID-19 is supposed to be the expression of an acute pulmonary infection with high viral loads. On the other hand, PIMS is conceived as a post-infectious syndrome with an exacerbated immune activation (9, 25, 26). Regarding physiopathology, some studies have identified different immunological profiles between pediatric acute COVID-19 and PIMS (27, 28). However, these studies fail to include groups of patients with severe-acute COVID-19 and patients with severe coexisting comorbidities, in contrast to our study.

Although ours was a small sample to make any inference, it supports the increasing awareness about the existence of different clinical phenotypes within positive PIMS criteria. Patients included in our cluster 1 were characterized by the presence of rash or gastrointestinal symptoms, with higher levels of some inflammatory markers. Custer 2 cases had a clinical presentation with predominant respiratory manifestations as described in severe acute COVID-19.

Some researchers have tried to define subgroups within patients who fulfill PIMS criteria. We tested several LCA-derived models using factors described in previous attempts of classification (14–16). Our final model includes only three factors (i.e., rash, gastrointestinal involvement, and severe respiratory involvement) to have a good balance between fit and parsimony with our small sample size. Godfred-Cato et al. (15), and later Geva et al. (16), identified three classes of patients: (1) a seriously ill group with significant cardiovascular involvement, multiple organ dysfunction, a pronounced elevation of inflammatory markers with a tendency of positive serology for SARS-CoV-2, (2) a group with predominantly respiratory involvement and high RT-PCR positivity rate that resembles our cluster 2 group and (3) a group with predominantly Kawasaki-like mucocutaneous findings. Meanwhile, Flood et al. also identified three PIMS phenotypes using cluster analysis: (1) PIMS with Kawasaki disease-like presentation, (2) PIMS with findings similar to Kawasaki disease and shock and (3) PIMS without any of these features (14). Accordingly, consensus-derived clinical recommendations have been generated (29). Of note, Flood et al. specified very high cutting points of laboratory and clinical signs to qualify as a PIMS, thus getting a more homogeneous sample and with higher levels of inflammation than we did. This circumstance and a bigger sample size let them classify cases in more categories and with subtler differences than we did.

In all three cluster analyses, coronary artery aneurysms were more frequent in groups with Kawasaki-like features and higher levels of inflammatory markers, which corresponds to cluster 1. Notably, none of the five aneurysm cases in our sample had serious underlying diseases and all of them belonged to cluster 1 (Mucocutaneous/gastrointestinal predominant symptoms).

The role of inflammation in the spectrum of severe illness associated with SARS-CoV-2 infection should be deeply studied, including its role in myocardial dysfunction since it is a frequent complication both in adults with COVID-19 and children with PIMS (30, 31). The resemblance of PIMS with Kawasaki disease and the notion of a dysregulated immune activation has led to the utilization of a similar therapeutic approach with intravenous immune globulin and glucocorticoids (32). Besides, it suggests the possible effectiveness of immune modulators such as tumor necrosis factor, interleukin-1, and interleukin-6 inhibitors.

A limitation of this study, as in others, is the method for identification of included cases for analysis, and which depended on spontaneous reports by treating clinicians. Besides, the secondary data source has the pitfall of lack of some data in the clinical files. It would have been desirable to have had the Ct’s and SARS-CoV-2 serology for every case as well as myocardial damage biomarkers. In the future, a prospective multicentric cohort study with clear-cut criteria for subclassification of every patient fulfilling the PIMS broad clinical criteria after SARS-CoV-2 infection would provide more information on the outcomes and possible associated factors.

## Conclusion

The epidemiology of PIMS cases observed in our center is comparable to those reported elsewhere, although our study included a high proportion of patients with an acute respiratory phenotype. Coronary aneurysms were uncommon and usually present in previously healthy patients with rash or gastrointestinal symptoms.

The death rate paralleled previous reports and occurred in patients with severe underlying diseases.

This study agrees with other researchers that in the context of case definition criteria for COVID-19, at least two groups of patients can be identified according to their clinical presentation. Standardized criteria for subclassification of PIMS cases need to be developed, since different phenotypes might have different pathophysiology, different outcomes, and possibly require different management. Clinical trials are needed to evaluate therapeutic interventions for different PIMS phenotypes.

## Data Availability Statement

The original contributions presented in the study are included in the article/supplementary material, further inquiries can be directed to the corresponding author/s.

## Ethics Statement

The studies involving human participants were reviewed and approved by Hospital Infantil de Mexico Federico Gomez Ethics Committee. Written informed consent from the participants’ legal guardian/next of kin was not required to participate in this study in accordance with the national legislation and the institutional requirements.

## Author Contributions

RG, MM, RJ-J, VO, and CD-B collected the data. HG coordinated the research team. MC-P and NG performed the data analysis and wrote the first draft of the manuscript. All authors contributed to the study plan and design, reviewed, and approved the final manuscript.

## Conflict of Interest

The authors declare that the research was conducted in the absence of any commercial or financial relationships that could be construed as a potential conflict of interest.

## Publisher’s Note

All claims expressed in this article are solely those of the authors and do not necessarily represent those of their affiliated organizations, or those of the publisher, the editors and the reviewers. Any product that may be evaluated in this article, or claim that may be made by its manufacturer, is not guaranteed or endorsed by the publisher.
